# Fitness of F1 hybrids between 10 maternal wild soybean populations and transgenic soybean

**DOI:** 10.1007/s11248-020-00230-x

**Published:** 2021-01-05

**Authors:** Jin Yue Liu, Ze Wen Sheng, Yu Qi Hu, Qi Liu, Sheng Qiang, Xiao Ling Song, Biao Liu

**Affiliations:** 1 Weed Research Laboratory, College of Life Sciences, Nanjing Agricultural University, Nanjing, 210095 Jiangsu Province China; 2 Ministry of Ecology and Environment, Nanjing Institute of Environmental Sciences, Nanjing, 210042 People’s Republic of China

**Keywords:** Transgenic soybean, Wild soybean, F1 hybrid, Fitness, Gene flow

## Abstract

The releasing of transgenic soybeans (*Glycine max* (L.) Merr.) into farming systems raises concerns that transgenes might escape from the soybeans via pollen into their endemic wild relatives, the wild soybean (*Glycine soja* Sieb. et Zucc.). The fitness of F1 hybrids obtained from 10 wild soybean populations collected from China and transgenic glyphosate-resistant soybean was measured without weed competition, as well as one JLBC-1 F1 hybrid under weed competition. All crossed seeds emerged at a lower rate from 13.33–63.33%. Compared with those of their wild progenitors, most F1 hybrids were shorter, smaller, and with decreased aboveground dry biomass, pod number, and 100-seed weight. All F1 hybrids had lower pollen viability and filled seeds per plant. Finally, the composite fitness of nine F1 hybrids was significantly lower. One exceptional F1 hybrid was IMBT F1, in which the composite fitness was 1.28, which was similar to that of its wild progenitor due to the similarities in pod number, increased aboveground dry biomass, and 100-seed weight. Under weed competition, plant height, aboveground dry biomass, pod number per plant, filled seed number per plant, and 100-seed weight of JLBC-1 F1 were lower than those of the wild progenitor JLBC-1. JLBC-1 F1 hybrids produced 60 filled seeds per plant. Therefore, F1 hybrids could emerge and produce offspring. Thus, effective measures should be taken to prevent gene flow from transgenic soybean to wild soybean to avoid the production F1 hybrids when releasing transgenic soybean in fields in the future.

**Supplementary information:**

The online version of this article (10.1007/s11248-020-00230-x) contains supplementary material, which is available to authorized users.

## Introduction

The cultivation area of transgenic soybeans have considerably increased since their commercial release in 1996. In 2018, transgenic soybeans were planted on 95.9 million hectare, reaching their highest level of adoption worldwide, covering 50% of the global biotech crop area, and 78% of the global soybean area (James 2019). Herbicide resistance, individually or stacked with insect resistance, has consistently been used as a dominant trait in soybeans. In 2017, herbicide-resistant soybeans accounted for 77% of the global biotech soybean area (James 2018). Although transgenic soybeans have not yet been commercially released in China, 17 transgenic soybeans were awarded safety certificates to allow them to be imported as raw material for processing from 2004, and 16 of these soybeans remain valid (http://www.moa.gov.cn/ztzl/zjyqwgz/spxx/). Since 2012, 80% of consumed soybeans have been imported, reaching 88.03 million tons in 2018 (http://data.stats.gov.cn), most of which were herbicide-resistant soybeans. Meanwhile, domestic transgenic soybeans have also been developed under governmental support. On January 21, 2020, China’s Ministry of Agriculture issued a biosafety certificate for production and field planting in the south of China of the glyphosate-resistant transgenic soybean SHZD32-01 developed by Shanghai Jiaotong University, representing a first step toward commercialized production of transgenic soybeans in China.

The release of transgenic soybeans to farming systems raises great concern that transgenes might escape from GM soybeans via pollen into their endemic wild relative, the wild soybean (*Glycine soja* Sieb. et Zucc.) (Kuroda et al. 2006; Yoshimura et al. 2011; Goto et al. 2017). Wild soybean (G genome, 2n = 40), the same genus as the cultivated soybean (*Glycine max* (Linn.) Merr.), is the progenitor of soybean and is extensively distributed in the Far East of Russia, the Korean peninsula, China, and Japan (Wang and Takahata 2007). Wild soybeans are an integral part of soybean genetic resources and are important for research on the origin and evolution of soybeans (Stupar 2010; Li et al. 2009, 2010; Akpertey et al. 2014). In China, wild soybeans are widely geographically distributed, almost in all soybean growing areas except for Hainan province, and they grow in diverse habitats even around farmland (Dong et al. 2001; Wang et al. 2017). Introgression from transgenic soybeans to wild soybeans may be happening.

Cultivated and wild soybeans share a common gene pool and can be reciprocally crossed resulting in fertile offspring without genetic isolation (Wang and Li 2011). The two species usually have a wide outcrossing rate of 2.4–37.4% in wild soybeans (Kiang et al. 1992; Fujita et al. 1997; Ohara and Shimamoto 2002; Kuroda et al. 2006), and extremely high values from 21.2 to 66.4% based on nuclear and chloroplast microsatellite variations (He et al. 2012). Furthermore, the most direct and convincing evidence that confirmed the natural occurrence of introgression between wild and cultivated soybeans was the natural F1 hybrids between maternal wild and paternal cultivated soybeans generated from original seed samples collected from wild soybean populations (Wang et al. 2010; Wang and Li 2011).

The first step for transgene escape is spontaneous hybridization between a transgenic crop and a compatible wild relative. Direct evidence for outcrossing from transgenic to wild soybeans has been reported in both Japan (Nakayama and Yamaguchi 2002; Mizuguti et al. 2009, 2010) and China (Chen et al. 2006; Liu et al. 2008, 2012, 2020). In these reports, different highest outcrossing rates were reported: 0.73% (Nakayama and Yamaguchi 2002), 0.19% (Liu et al. 2020), and 0.097% (Mizuguti et al. 2010), due to different innate factors (reproductive compatibility, sympatry, and flowering synchrony), weather, and environmental conditions.

Besides the initial crop–weed hybridization, the likelihood of transgenes or other alleles spreading from crops to related wild populations depends on the fitness of the first and successive generations of hybrids (Hauser et al. 1998; Gueritaine et al. 2002; Jenczewski et al. 2003; Lu and Snow 2005; Kuroda et al. 2006; Guan et al. 2015; Kan et al. 2015). Fitness is reflected by vegetative and reproductive growth indicators and determines whether the hybrids can survive and establish (Warwick et al. 2009). Variation in fitness is expected across subsequent hybrid generations due to recombination between the genomes of wild and cultivated species (Kling 1996; Chèvre et al. 1997; Ammitzbøll 2005; Allainguillaume et al. 2006). The fitness of early hybrids relative to their parents determines transgene establishment in wild relatives (Jenczewski et al. 2003). Therefore, F1 hybrid viability/fertility should represent a bottleneck for transgene escape. For the alleles in one population to introgress to another, the initial hybrid generations must be viable and at least partially fertile (Chapman and Burke 2006).

The fitness of hybrids might vary widely, depending on the parental genotype, testing environment, and their interaction (Campbell and Waser 2001; Johnston et al. 2001; Johannessen et al. 2006; Whitney et al. 2006; Yang et al. 2011; Liu et al. 2016; Huang et al. 2019). Therefore, it is important to evaluate the fitness of hybrids of transgenic crops and wild relatives under different conditions. There is a dearth of previous research on the fitness of F1 hybrids obtained from wild and cultivated soybeans, including transgenic soybeans. Guan et al. (2015) measured the F1 hybrids of two wild soybeans and glyphosate-resistant soybean (AG5601) in a greenhouse, and found that two F1 hybrids had similar aboveground biomass, the same or 36% fewer pods, and the same or 54% fewer seeds per plant compared with their wild relatives. However, the 100-seed weight of two F1 hybrids was three times that of their respective wild relatives. Kan et al. (2015) reported on F1 hybrid performance obtained from four wild soybeans and glyphosate-resistant transgenic soybean. They found that four F1 hybrids performed differently compared with their wild relatives, e.g., differences were observed in pod number per plant, one of the four F1 hybrids produced 65% more, two produced 39% and 56% fewer, and the last one had a similar number of pods to its wild relative.

Although studies have shown that F1 hybrids between wild and cultivated soybeans exhibited performance that was lower, similar, or higher compared with that of their wild relatives for some traits, these results still do not fully reflect the potential ecological risks of cultivating GM soybeans in China because few populations of wild soybeans have been studied (Guan et al. 2015; Kan et al. 2015). In previous study, we collected 10 wild soybean populations from six provinces. The crossed seeds were obtained between these wild soybeans as maternal plants and transgenic glyphosate-resistant soybean as paternal plants (Hu et al. 2020). The average podding rates and the average filled seed number per pod of 10 wild soybean populations crossed with transgenic soybean ranged from 8.85–17.97%, and from 0.20 to 0.48, respectively. In the current study, the performance of F1 hybrids was measured in a net house without competition with weeds for 10 F1 hybrids as well as one F1 hybrid under competition with weeds. We aimed to predict the risk and consequences of gene flow from transgenic soybean to different wild soybeans and the potential risk without competition and under competition with weeds.

## Materials and methods

The glyphosate-resistant (GR) soybeans (T14R 1251–70) were provided by the National Soybean Improvement Center of Nanjing Agricultural University. The GR soybean, contains one single-copy *cp4-epsps*, was obtained by *Agrobacterium*-mediated co-transformation of the receptor soybean NJR44-1, which is an elite line bred by the National Soybean Improvement Center of Nanjing Agricultural University. The GR soybean withstands 3600 g a.i. ha^−1^ 41% glyphosate isopropylammonium AS (Roundup Ultra; Monsanto, St. Louis, MO, USA).Ten wild soybean populations were collected from six provinces, namely Heilongjiang, Jilin, Liaoning, Hebei, Henan, and Jiangsu, and the Inner Mongolia Autonomous Region during 2015–2016 (Table 1). Crossed seeds were obtained by artificial hybridization of wild soybeans as the maternal plants crossed with transgenic soybean as the paternal plants from 2016 to 2017 (Hu et al. 2020). The crossed seeds were harvested from different mother plants, mixed, and then stored at 4 °C until further use. Experiments were conducted in a greenhouse and net house at the Pailou Experimental Farm (118°37′E, 32°02′N), Nanjing Agricultural University, China, from 2017 to 2019.

**Table 1:** Information of wild soybeans used in the experiments

Collecting site	Population number	Latitude and longitude
Harbin City, Heilongjiang Province	HLJHRB-1	N46°06′34″, E127°21′43″
Harbin City, Heilongjiang Province	HLJHRB-2	N46°04′44″, E127°23′02″
Baicheng City, Jilin Province	JLBC-1	N45°31′23″, E124°17′19″
Baicheng City, Jilin Province	JLBC-2	N45°31′20″, E124°19′57″
Tieling City, Liaoning Province	LNTL	N42°17′28″, E123°51′47″
Shenyang City, Liaoning Province	LNSY	N41°32′41″, E123°27′29″
Baotou City, Inner Mongolia Autonomous Region	IMBT	N40°37′37″, E109°54′14″
Handan City, Hebei Province	HBHD	N36°38′59″, E114°36′35″
Shangqiu City, Henan Province	HNSQ	N34°22′21″, E115°40′18″
Changzhou City, Jiangsu Province	JSCZ	N31°37′13″, E119°29′53″

### Seed sowing and emergence

At the beginning of May of the next year (the second year for IMBT F1 and HNSQ F1) after crossed seeds were obtained by artificial hybridization, 20 filled seeds of each wild soybean population and transgenic soybean were selected from the 10 mother plants (two seeds from each plant). Then single filled seed of each wild soybean population and transgenic soybean were sown at 1 cm depth in individual pots (7 cm diameter, 7.5 cm height) previously filled with a mixture of wasteland soil and organic cultivated soil (Green Island Horticultural Development Center, Zhenjiang, China) at a 1:1 (v/v) ratio. The filled crossed seeds were also selected and sown using the same method. Owing to limited filled crossed seeds obtained after artificial hybridization, six filled crossed seeds of JSCZ, HNSQ, and IMBT, 10 filled crossed seeds of LNTL, 15 filled crossed seeds of HLJHRB-2, JLBC-2, LNSY, and HBHD, 20 filled crossed seeds of HLJHRB-1, and 24 filled crossed seeds of JLBC-1 were randomly selected and each seed sown directly into individual plastic pots. The experiments were replicated five times. Number of wild soybeans and crossed seeds sown in the experiments are disclosed in detail in supplementary Table 1.

Pots were laid out in a completely randomized design in the same replicate in the greenhouse. Wild soybean seed coats were sturdy and durable under natural state, so the embryo-dorsal seed coats (on the opposite of hilum) of wild soybeans and crossed seeds were carefully nicked with a razor blade prior to sowing (seed coat was broken but the internal structure of the seeds was undamaged), to break the limit of imperviousness of the seed coat. Experimental pots were exposed to natural light and photoperiods, approximately 13 light/11 dark, and the temperature fluctuated from 22 to 28 °C during the experiment. The pots were watered as needed and hand weeded.

Emergence number of transgenic soybeans, wild soybeans, and crossed seeds was recorded when the cotyledon emerged from the soil and completely turned green approximately 2 weeks after sowing. Emergence rate (%) = (emergence number / number of seeds sown) × 100. The length and width of cotyledons and true leaves were measured using vernier calipers (Shanghai Meinaite Industrial Co., Ltd, China) when the first trifoliolate leaf was not completely spread. The size of cotyledons and true leaves was calculated as the length Χ width of cotyledons + the lenght Χ width of true leaves.

### F1 hybrids confirmed by testing *cp4-epsps* gene

All emerging F1 seedlings were tested by PCR to confirm whether they contained the *cp4-epsps* gene. The DNA of each F1 seedling was extracted with a Plant Genomic DNA Kit [Tiangen Biotech (Beijing) Co., Ltd.], according to the manufacturer's instructions. A set of primer pairs for PCR analysis was designed using the sequence of the *cp4-epsps* gene included in the transgenic soybeans with Primer 6.0 Software (primer P1: 5′GGCACAAGGGATACAAACCC3′; primer P2: 5′ACCGCCGAACATGAAGGAC3′).

Each PCR reaction involved a 20-µL reaction solution containing 10 µL of Premix Taq Version 2.0 plus dye [Treasure Biological Engineering (Dalian) Co., Ltd], 6 µL of double distilled water, 1 µL of forward primer (10 µM), 1 µL of reverse primer (10 µM), and 2 µL of 20–30 ng/µL genomic DNA. PCR amplification was performed on a Whatman Biometra TGRADIENT Thermocycler at 95 °C for 5 min for the initial denaturation, 35 cycles of denaturation at 95 °C for 30 s, annealing at 58 °C for 50 s, elongation at 72 °C for 1 min, and a final extension at 72 °C for 10 min. Amplified DNA products were separated on 1% agarose gels at 120 V for 30 min, stained with 10,000 × SolarRed, and visualized under UV light. Wild and transgenic soybeans were used as negative and positive controls, respectively.

### Seedling transplanting and variables measured

#### Without weed competition

At least 15 plants uniform in size of wild soybeans, transgenic soybeans (the transplanting number were the same with wild soybean in the same year), and 10–29 F1 plants (Table 2) with the *cp4-epsps* gene were transplanted individually into pots with holes at the bottom (23 diameter cm, 25 cm height), containing the same growth media as described previously when the second trifoliolate leaf spread completely. On the third day after transplanting, a bamboo pole (2 diameter cm, 200 cm height) was inserted into the pots with wild soybeans and F1 seedlings for the plants to climb. Pots were watered and hand-weeded as needed. No chemicals were applied during the experiment. Seedlings were grown under natural conditions exposed to natural light (approximately 14–11 light) and temperature (approximately 15–35 °C) from the date of transplanting to harvesting (from the end of June to the end of November). Adjacent pots were separated by 60 cm. Pots were laid out in a completely randomized design in the net house, and no sexually compatible Leguminosae species were present for a 100-m radius around the experiment. The plant height was measured from the top of the plant to the cotyledonary node when the third trifoliolate leaf spread completely.

**Table 2:** Number of wild soybeans and F1 hybrids transplanted in the experiments

Wild soybean	Number			F1 Hybrid	Number		
	2017	2018	2019		2017	2018	2019
HLJHRB-1	30	–	–	HLJHRB-1 F1	29	–	–
JLBC-1	30	–	–	JLBC-1 F1	25	–	–
LNTL	30	–	–	LNTL F1	15	–	–
JSCZ	30	–	–	JSCZ F1	13	–	–
HLJHRB-2	–	20	–	HLJHRB-2 F1	–	19	–
JLBC-2	–	20	–	JLBC-2 F1	–	10	–
LNSY	–	20	–	LNSY F1	–	13	–
HBHD	–	20	–	HBHD F1	–	20	–
IMBT	–	–	15	IMBT F1	–	-	11
HNSQ	–	–	15	HNSQ F1	–	-	13

The other fitness components were measured as follows. Pollen viability was tested at the full flowering stage. Pollen was collected from nascent flowers at 7–8 am, and the in vitro pollen germination rate at 100 min was tested according to the method described by Liu and Liu (2018). At least 50 pollen grains from five flower buds on each of the one to three plants for wild soybean population, transgenic soybean and F1 plants were used as one replicate, and a total of nine replicates were assessed each time. Finally, the in vitro pollen germination rate was calculated as follows: (pollen germinated/pollen observed) × 100. When pollen tube length was twice pollen grain length, it was considered to have germinated.

When 100% pods darkened (harvest maturity), each individual plant was separately harvested (cut from cotyledonary node). Each plant was sun dried to a constant weight and the aboveground dry biomass was weighed. The number of pods of each harvested plant was counted. All seeds were hand-peeled from the pods. Then, the number of filled seeds was counted for each plant. After being sun dried for 10 days in a greenhouse, 100 filled seeds were randomly counted from 10 plants and weighed for each wild soybean, transgenic soybean, and F1 hybrid.

#### With weed competition

On the same day of sowing wild soybeans and JLBC-1 F1 hybrids in the experiment without weed competition in 2017, 0.5 g of seeds each from *Setaria viridis* (L.) Beauv*.*, *Digitaria sanguinalis* (L.) Scop., and *Echinochloa colona* (L.) Link., 0.25 g of seeds from *Eleusine indica* (L.) Gaertn., and 0.2 g of seeds from *Amaranthus retroflexus* L. were well mixed and then sown evenly on the surface of pots with holes in the bottom (52 diameter cm, 35 cm height). The pots contained the same media as those in the experiment without competition. These pots were watered and cultured in the net house.

We imposed interspecific competition by transplanting JLBC-1 F1 hybrids and its progenitors, JLBC-1 and transgenic soybeans, into recently established stands of weeds in 2017. Fifteen uniformly sized wild soybean of JLBC-1, transgenic soybean, and JLBC-1 F1 plants with the *cp4-epsps* gene were individually transplanted into the pots with weeds. The methods for measuring performance variables, including plant height, aboveground dry biomass, pod number per plant, and filled seed number per plant, were the same as those used in the experiment without weed competition.

## Statistical analysis

Separation of the means of each measured variable was performed following the method described by Liu et al. (2016) using SPSS II 25.0 Software (IBM). Without weed competition, the means of every measured variable of wild soybeans and F1 hybrids were calculated. The average of each variable of wild soybeans was defined as ‘1′, and the ratio of the variables between each F1 hybrid and its respective wild soybean was defined as the relative variable value. Composite fitness across the history of the whole cycle was the mean of the relative variable estimates for the entire cycle from vegetative to mature stages, including emergence rate, size of cotyledons and true leaves, plant height, aboveground dry biomass, pollen viability, pod number per plant, filled seeds per plant, and 100-seed weight.

The means of each variables and composite fitness for the F1 hybrids and the respective wild soybeans without weed competition were separated using a t-test for independent samples.

With weed competition, the performance of all variables among F1 hybrids, wild soybeans, and transgenic soybean were separated using Duncan’s multiple range test. The composite fitness of JLBC-1, its F1 hybrid and transgenic soybean was the mean of the relative variable estimates for plant height, aboveground dry biomass, pod number per plant, filled seeds per plant, and 100-seed weight without weed competition compared with those under weed competition. The difference in composite fitness of JLBC-1, its hybrids and transgenic soybean without and with weed competition were separated using a t-test for independent samples.

## Results

### Emergence rate of crossed seeds

**Fig. 1:**
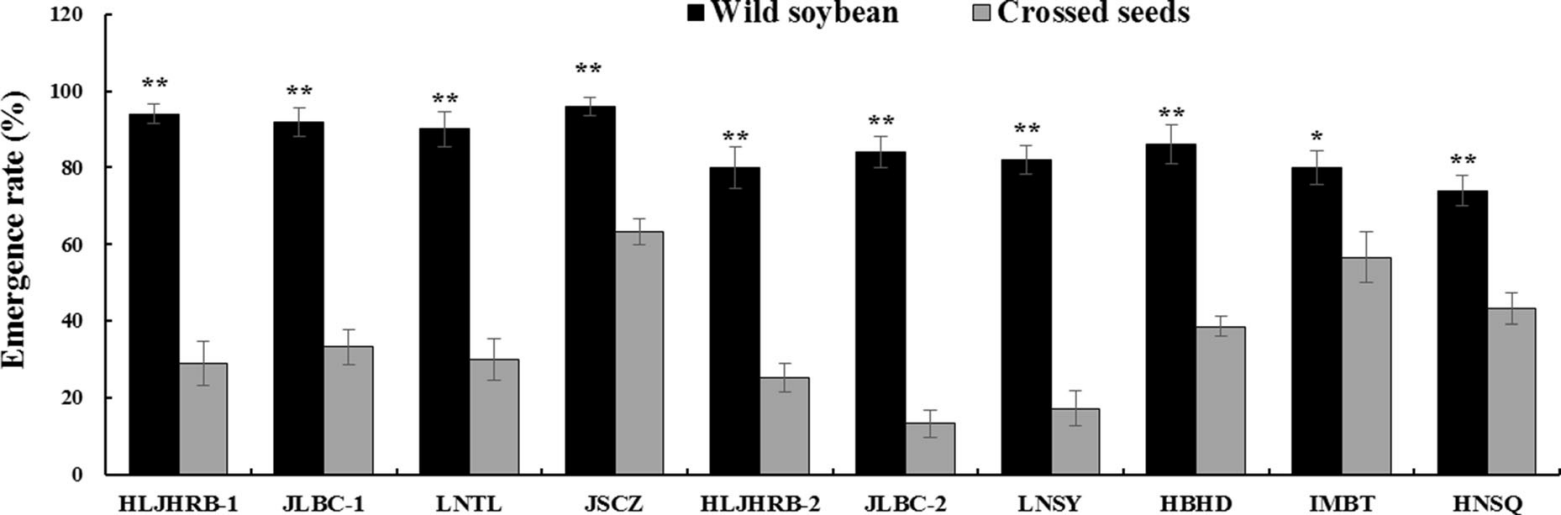
Emergence rate of crossed seeds obtained from wild soybeans and transgenic soybean. Data were shown as the mean ± SE. * and ** indicate significant differences (*P* < 0.05 and *P* < 0.01, respectively) between wild soybean and its crossed seeds with transgenic soybean separated using a t-test for independent samples

Wild soybeans displayed an emergence rate of 74.00–96.00%. The emergence rate of crossed seeds was 23.33–70.67% less than that of their respective wild soybeans (*P* < 0.01 for nine F1 hybrids except for IMBT, it’s was *P* < 0.05) (Fig. 1).

### Glyphosate-resistant gene transmission

**Fig. 2:**
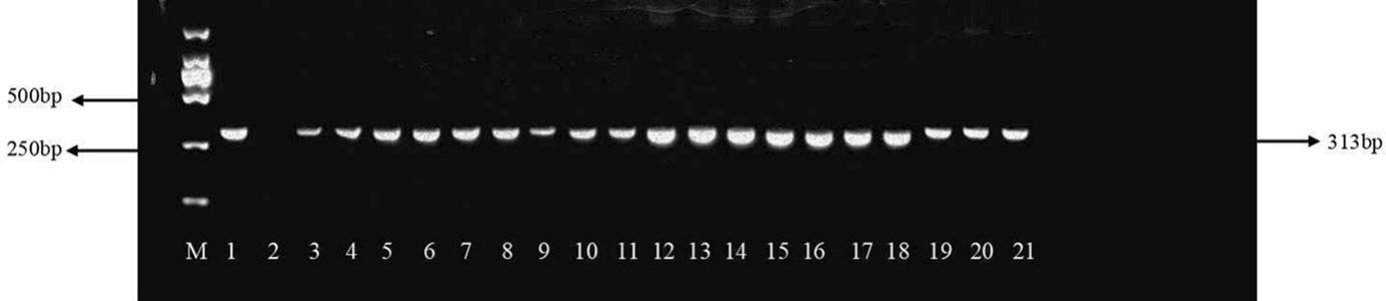
PCR amplification of *cp4psps* gene fragment from the F1 hybrids between wild soybean HLJHRB-2 and transgenic glyphosate-tolerant soybean. M: marker DL2000; 1: Glyphosate resistant transgenic soybean; 2: Wild soybean HLJHRB-2; 3–21: F1 hybrids of HLJHRB-2

The glyphosate-resistant gene PCR amplification tests showed that the *cp4-epsps* (313 bp) fragments from the transgenic soybeans were highly conserved in the F1 hybrids (Fig. 2, taking F1 hybrids of HLJHRB-2 as an example).

### Without weed competition

All wild soybean, transgenic soybean, and F1 hybrid plants grew and flowered vigorously, and no plants died from transplanting to harvest. The morphology of nine F1 hybrids was twinning, and black pod, small darker seeds traits from wild soybeans. Seeds size and color of IMBT F1 hybrid were intermediate between those of the parents. We compared the performances of F1 hybrids and their wild soybeans in Table 3. The performance of transgenic soybean in 3 years is shown in Supplementary Table 2.

**Table 3:** Performance of F1 hybrids and their wild soybeans without weed competition (mean ± SE)

	Length of cotyledons	Width of cotyledons	Length of true leaves	Width of true leaves	Plant height	Aboveground dry biomass	Pollen viability	Pod number/plant	Filled seeds number/plant	100-seed weight
HLJHRB-1	10.82 ± 0.09**	5.92 ± 0.07**	17.90 ± 0.24**	12.16 ± 0.16**	20.54 ± 0.41**	79.09 ± 3.31*	82.89 ± 1.50**	219.03 ± 8.39**	619.80 ± 31.74**	1.15 ± 0.01**
HLJHRB-1 F1	7.92 ± 0.09	4.25 ± 0.07	14.35 ± 0.32	8.32 ± 0.19	8.55 ± 0.38	70.05 ± 2.83	68.22 ± 1.13	184.24 ± 5.59	307.34 ± 16.96	1.06 ± 0.02
JLBC-1	10.81 ± 0.09**	6.73 ± 0.10**	17.84 ± 0.31**	13.20 ± 0.22**	18.27 ± 0.67**	67.73 ± 1.77**	86.12 ± 1.16**	179.77 ± 9.74**	511.77 ± 30.53**	1.73 ± 0.03**
JLBC-1 F1	6.28 ± 0.07	3.43 ± 0.03	10.85 ± 0.18	7.33 ± 0.14	13.92 ± 0.75	55.24 ± 2.21	60.15 ± 1.57	90.48 ± 5.50	117.28 ± 11.19	1.49 ± 0.02
LNTL	10.13 ± 0.12ns	5.53 ± 0.08ns	17.10 ± 0.23ns	12.05 ± 0.17ns	23.23 ± 0.50**	113.63 ± 4.38**	76.78 ± 1.36**	398.43 ± 11.78**	831.20 ± 29.35**	1.15 ± 0.03ns
LNTL F1	10.12 ± 0.15	5.52 ± 0.07	17.07 ± 0.22	11.74 ± 0.18	6.61 ± 0.43	43.93 ± 2.03	69.80 ± 1.67	89.40 ± 5.05	73.40 ± 4.40	1.09 ± 0.02
JSCZ	10.35 ± 0.14**	5.73 ± 0.08**	18.88 ± 0.23**	13.18 ± 0.14**	47.50 ± 0.69**	82.16 ± 2.66ns	89.90 ± 1.64**	307.43 ± 12.97ns	764.23 ± 34.93**	2.21 ± 0.03**
JSCZ F1	7.28 ± 0.11	4.11 ± 0.09	10.79 ± 0.28	7.93 ± 0.18	16.77 ± 1.17	87.38 ± 6.28	73.65 ± 1.26	268.46 ± 24.40	405.23 ± 61.55	1.99 ± 0.02
HLJHRB-2	9.99 ± 0.20ns	5.74 ± 0.08**	14.73 ± 0.27**	9.96 ± 0.24**	18.61 ± 0.47**	36.84 ± 2.36**	85.36 ± 1.89**	165.80 ± 11.71**	356.35 ± 26.84**	1.07 ± 0.03ns
HLJHRB-2 F1	9.59 ± 0.13	5.02 ± 0.07	13.52 ± 0.29	8.64 ± 0.27	9.78 ± 0.45	23.88 ± 1.02	66.58 ± 4.36	113.84 ± 6.83	154.16 ± 14.87	1.03 ± 0.03
JLBC-2	10.73 ± 0.11**	6.40 ± 0.08**	19.78 ± 0.29**	15.20 ± 0.24**	21.50 ± 0.65**	49.08 ± 3.11**	95.93 ± 0.80**	221.10 ± 16.65ns	471.40 ± 41.27**	1.21 ± 0.03ns
JLBC-2 F1	9.11 ± 0.25	5.32 ± 0.15	14.92 ± 0.44	11.19 ± 0.39	13.42 ± 0.86	34.92 ± 1.34	64.92 ± 1.17	202.50 ± 15.83	283.30 ± 22.43	1.16 ± 0.03
LNSY	9.49 ± 0.16**	5.45 ± 0.10**	14.75 ± 0.39**	10.77 ± 0.27**	20.34 ± 0.64**	68.76 ± 3.14**	90.25 ± 1.34**	423.35 ± 20.90ns	803.15 ± 36.38**	1.16 ± 0.03*
LNSY F1	8.47 ± 0.20	4.75 ± 0.14	12.70 ± 0.32	8.90 ± 0.36	13.33 ± 0.90	50.18 ± 3.34	49.57 ± 1.47	374.69 ± 37.90	392.62 ± 42.18	1.06 ± 0.03
HBHD	10.66 ± 0.11**	6.00 ± 0.10**	17.87 ± 0.42**	12.91 ± 0.31**	17.93 ± 0.55**	78.92 ± 3.98**	71.13 ± 1.89**	494.80 ± 38.40ns	893.80 ± 76.28**	1.33 ± 0.02**
HBHD F1	9.30 ± 0.22	5.32 ± 0.13	15.43 ± 0.45	11.00 ± 0.42	11.03 ± 0.44	59.23 ± 3.55	60.91 ± 1.81	397.55 ± 29.12	508.45 ± 36.79	1.23 ± 0.02
IMBT	12.10 ± 0.20**	7.31 ± 0.09**	20.04 ± 0.42**	15.46 ± 0.39**	16.05 ± 0.34**	20.81 ± 1.38	82.71 ± 0.56**	206.00 ± 15.50ns	497.27 ± 44.14**	1.55 ± 0.03
IMBT F1	10.12 ± 0.14	6.59 ± 0.11	17.57 ± 0.23	12.86 ± 0.15	13.58 ± 0.53	39.62 ± 4.00**	76.52 ± 1.26	188.82 ± 19.07	317.09 ± 43.14	5.52 ± 0.22**
HNSQ	10.21 ± 0.23ns	5.87 ± 0.12ns	17.19 ± 0.34ns	13.18 ± 0.22**	18.02 ± 0.35ns	47.50 ± 1.39*	90.34 ± 0.95**	313.13 ± 9.01**	740.80 ± 25.80**	1.93 ± 0.02**
HNSQ F1	10.31 ± 0.10	6.03 ± 0.11	16.72 ± 0.23	12.12 ± 0.20	17.88 ± 0.34	41.47 ± 2.07	82.17 ± 1.16	243.23 ± 9.10	571.00 ± 24.17	1.77 ± 0.03

*and ** Indicate significant differences (*P* < 0.05 and *P* < 0.01, respectively) between F1 hybrid and its wild progenitor separated using a t-test for independent samples. NS means no significant differences (*P* > 0.05) between F1 hybrid and its wild progenitor separated using a t-test for independent samples

#### Performance in the vegetative stage

Most F1 hybrids had cotyledons that were significantly smaller in both length and width as were the true leaves (*P* < 0.01). However, LNTL F1 hybrids exhibited cotyledons and true leaves of similar sizes. HNSQ F1 hybrids had similar cotyledons size and true leaves length. HLJHRB-2 F1 hybrids had cotyledons of similar length. Nine of the 10 F1 hybrids were significant shorter at 71.55–15.39% (*P* < 0.01) the height of their wild soybeans, except for HNSQ F1 hybrids, which were 17.88 cm tall, similar to HNSQ. Seven of the 10 F1 hybrids had significantly lower aboveground dry biomass (61.34–18.44%) than their respective wild parents (*P* < 0.01). HNSQ F1 and HLJHRB-1 F1 hybrids were 12.69 and 11.43% (*P* < 0.05) lower. IMBT F1 hybrids were an exception at 90.39% higher than its wild parent (Table 3).

#### Performance on reproductive stage

The pollen germination rates of wild soybeans ranged from 71.13% to 95.93% in the in vitro experiments at 100 min, while the pollen germination rates of F1 hybrids were 49.57–82.17%, which were significantly lower than those of their wild parents (*P* < 0.01). F1 hybrids of HLJHRB-1, JLBC-1, LNTL, HLJHRB-2, and HNSQ produced 15.88–77.56% fewer pods per plant than their wild progenitors (*P* < 0.01). JSCZ F1, JLBC-2 F1, LNSY F1, HBHD F1, and IMBT F1 produced 188.82–397.55 pods per plant, which was similar to the number produced by their wild parents.

All 10 F1 hybrids produced significantly fewer filled seeds per plant than their wild parents (*P* < 0.01) at 169.80–757.80. HNSQ F1, IMBT F1, JLBC-2 F1, and JSCZ F1 hybrids exhibited better performance, which difference with wild parents were 22.92, 36.23, 39.90, and 46.98%, respectively. The filled seed number of HLJHRB-1 F1, HLJHRB-2 F1, and LNSY F1 hybrids was approximately 50% of that of their wild parents. JLBC-1 F1 and LNTL F1 hybrids produced 77.08 and 91.17% fewer seeds than their wild parents.

Compared with their respective wild parents, LNTL F1, HLJHRB-2 and JLBC-2 F1 hybrids had no significant differences in 100-seed weight. In the other six F1 hybrids, 100-seed weight was significantly lower (7.52–13.87%) than that of their wild progenitors (*P* < 0.01 or 0.05). However, IMBT F1 hybrids had a large 100-seed weight (5.52 g), which was 256.13% greater than that of its wild parent. Besides larger seeds, the seed coat color of IMBT F1 hybrids turned to the color of transgenic soybean. Pictures of the seeds are shown in Supplementary Fig. 1.

#### Composite fitness

**Fig. 3:**
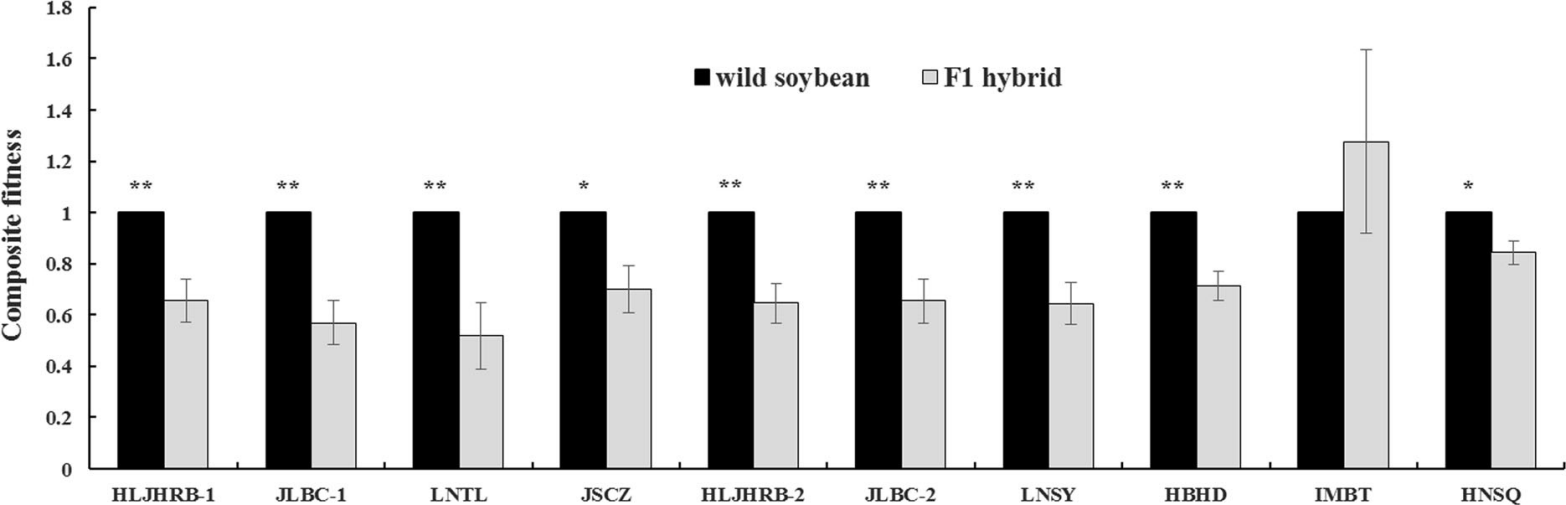
Composite fitness of F1 hybrids and their wild soybeans without weed competition. To estimate composite fitness, the variables of wild soybean were defined as ‘1′. Data were shown as the mean ± SE (n = 8). * and ** indicate significant differences (*P* < 0.05 and *P* < 0.01, respectively) between F1 hybrid and its wild progenitor separated using a t-test for independent samples

Nine of the 10 F1 hybrids remained less fit than their wild progenitors, with composite fitness ranging from 0.52 to 0.84. One exceptional F1 hybrid was IMBT F1 with 1.28 composite fitness, which was similar to that of its wild progenitor (*P* = 0.4590) (Fig. 3). It had a similar number of pods and an increased aboveground dry biomass and 100-seed weight compared with its wild progenitor.

### With weed competition

Weeds grew vigorously and similarly in each pot in the whole experiment. After being harvested, weeds occurred in JLBC-1 F1 hybrid and its progenitor wild soybean and transgenic soybean at 95.62, 98.16, and 103.27 g, but there was no significant difference in aboveground dry biomass. This implied that the interference of weeds on JLBC-1 F1 hybrid and its progenitors was similar.

Five plants of JLBC-1 F1 hybrids died at the earlier third trifoliolate leaf stage after transplanting. Two plants of JLBC-1 died at the later third trifoliolate leaf stage. JLBC-1 F1 hybrids performed much weaker compared with its wild and cultivated parents. The plant height,  aboveground dry biomass, pod number per plant, filled seeds number per plant, and 100-seed weight were lower by 29.70, 57.19, 53.22, 64.90, and 17.72%, respectively, than its wild progenitor JLBC-1 (*P* < 0.05). The plant height, aboveground dry biomass, and 100-seed weight were lower by 59.00, 83.55, and 90.97%, respectively, than its paternal parent transgenic soybean (*P* < 0.05). Meanwhile, JLBC-1 F1 produced 65.10% more pods per plant (*P* < 0.05) and 24.79% more filled seeds per plant than its paternal parent transgenic soybean (Table 4).

**Table 4:** Performance of transgenic soybean, wild soybean JLBC-1 and their F1 hybrid under weed competition (Mean ± SE)

Trait	Transgenic soybean	JLBC-1	JLBC-1 F1
Plant height (cm)	28.00 ± 1.20a	16.33 ± 0.69b	11.48 ± 0.59c
Aboveground dry biomass (g)	100.33 ± 3.40a	38.54 ± 2.79b	16.50 ± 1.74c
Pod number/plant	19.2 ± 1.94c	67.77 ± 5.30a	31.70 ± 3.64b
Filled seed number/plant	48.4 ± 5.16b	172.08 ± 18.40a	60.40 ± 6.88b
100-seed weight (g)	14.39 ± 0.12a	1.58 ± 0.04b	1.30 ± 0.04c

Different letters in the same row indicate significant differences among transgenic soybean, JLBC-1 and JLBC-1 F1 using Duncan’s multiple range test, *P* < 0.05

### Comparing performance with and without weed competition

**Fig. 4:**
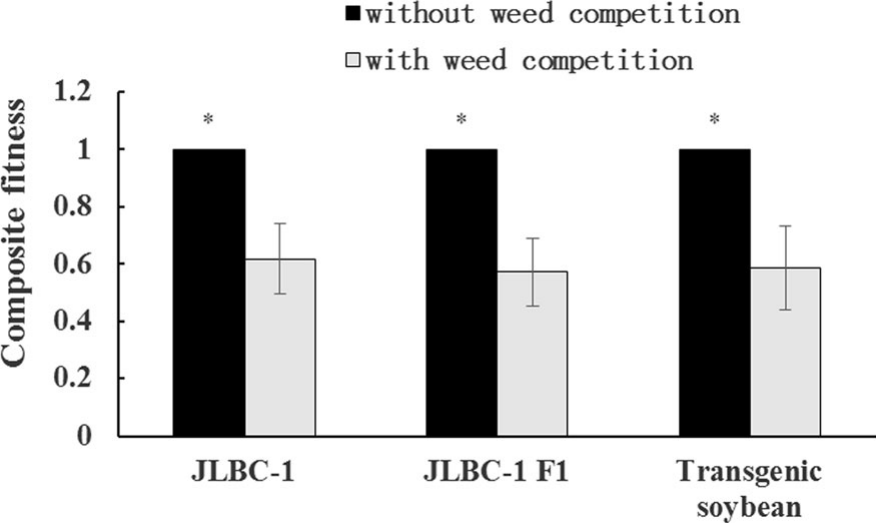
Composite fitness of JLBC-1 F1 hybrid and its parents with weed competition compared with those without weed competition. To estimate composite fitness, the variables without weed competition were defined as ‘1′. Data were shown as the mean ± SE (n = 5, Plant height, Aboveground dry biomass, Pod number/plant, Filled seed number/plant, and 100-seed weight). * indicate significant differences (*P* < 0.05) between composite fitness with weed competition compared with those without weed competition of F1 hybrid, its wild progenitor or transgenic soybean separated using a t-test for independent samples

Weed competition impacted the performance of JLBC-1 F1 hybrids and their wild and cultivated progenitor. JLBC-1 F1 hybrids decreased by 17.53, 64.96, 48.50 and 12.75% in plant height, pod number per plant, filled seeds per plant, and 100-seed weight, respectively, which did not have much more differences with those of its maternal wild soybean and paternal transgenic soybean. However, aboveground dry biomass of JLBC-1 F1 hybrids decreased by 70.13%, while its maternal and paternal plants decreased by 43.10% and 22.54% (Table 5). Finally, the composite fitness of JLBC-1, its F1 generation and transgenic soybean was 0.62, 0.57, and 0.58 compared with those without weed competition (Fig. 4).

**Table 5:** The decreased percentage (%) of JLBC-1 F1 hybrid and its progenitors performance under weed competition compared with those without weed competition

Traits	Transgenic soybean	JLBC-1	JLBC-1 F1
Plant height (cm)	10.00*	10.62	17.53
Aboveground dry biomass (g)	22.54*	43.10**	70.13**
Pod number/plant	77.09**	62.30**	64.96**
Filled seed number/plant	76.99**	66.38**	48.50**
100-seed weight (g)	21.28**	8.67**	12.75**

* and ** indicate significant difference of F1 hybrid, or its wild progenitor or transgenic soybean under weed competition compared with those without weed competition separated using a t-test for independent samples (*P* < 0.05 and *P* < 0.01)

## Discussion

### Emergence rate

The growth cycle of annual plants begins with the germination of seeds. Plant species with annual life cycles completely depend on seed germination (Calado et al. 2011). In our study, all crossed seeds obtained from maternal wild soybeans and transgenic soybeans emerged at 13.33–63.33%, indicating that these crossed seeds were viable. However, crossed seeds emerged at a lower rate than those of their wild progenitors. A lower emergence rate may be a factor restricting F1 hybrids from establishing populations. The reason may be lower seed viability rather than seed coat impermeability. Seed and embryo viability are associated. Although cultivated soybeans and wild soybeans carry similar genomes (GG, 2n = 40) (Singh and Hymowitz 1985, 1988), the meiotic aberrations, heteromorphic chromosome pairing for chromosomes 6 and 11, were observed in *G. max* × *G. soja* F1 hybrids (Singh and Hymowitz 1988). Moreover, wild soybean accessions were separated into two types on the basis of chromosome structure; 19% had normal chromosome structure, while 81% had chromosome interchange (Palmer et al. 1987). Chromosome interchanges resulted in bridges and multivalent formation during meiosis that reduce pollen and seed fertility in hybrids (Palmer et al. 2000). Therefore, the variable crossed seed viability should be due to cryptic structural differences between wild and cultivated soybeans as well as genotype differences in wild soybeans.

Hard seededness of wild soybeans is an adaptive character to survive for long periods in adverse wild environments and germinate at a favorable time. This characteristic in cultivated soybeans has been greatly reduced through artificial selection (Zhou et al. 2010; Vu et al. 2014). We did not evaluate the water permeability of crossed seeds because we obtained limited seeds after artificial hybridization. The crossed seeds could lose this favorable characteristic due to the influence of the transgenic soybean seed coat allele, which is responsible for the reduction in F1 hybrid fitness. The increased permeability in seeds obtained from wild soybeans and transgenic soybeans as well as offspring of F1 hybrids will not protect seeds against deterioration, and maintain viability in natural environments. From this point, F1 hybrids may exhibit lower fitness. However, fitness of F1 plants may be potentiality increased in further generation either by selfing or backcrossing to wild parents.

### Without competition

#### Plant height

Wild soybeans are herbaceous annual plants and the seeds fall from the parental plants, which die at the end of the growing season, and the seeds grow out from the soil surface either in growing or already established vegetation. This often results in severe competition for light with surrounding plants at early stages of their growth. Under this circumstance, taller plants at early stages of their growth could contribute to this competitiveness during the initial phases of plant establishment and growth (den Dubbelden and Verburg 1996). Taller plants are more competitive than shorter ones due to better light interception, which is directly associated with the photosynthetic activity of the plant (Cudney et al. 1991; Caton et al. 1999; Cousens et al. 2003a, b; West et al. 2010; Denison et al. 2010).

In the present study, we measured plant height at the third trifoliolate leaf stage, and found that nine of the 10 F1 hybrids were significant shorter (*P* < 0.05) than wild soybeans by 15.39–71.55%, except for HNSQ F1, which was similar to its wild progenitor. The results implied that most F1 hybrids had a disadvantage in plant height compared with maternal wild soybeans at the seedling stage. The results on plant height of F1 hybrids were similar to that of the previous study (Kan et al. 2015). Even though, the plant height of F1 hybrids may change in different environment. Therefore, further research should be conducted in different environment.

#### Aboveground dry biomass

Variation in the total biomass of annual plants at the end of the growing season reflects differences in resource capture and biomass production rate (Dovrat et al. 2019). High aboveground dry biomass implied more competitiveness. Nine of the 10 F1 hybrids had significantly lower aboveground dry biomass than their respective wild parents (*P* < 0.01). This result implied that these nine F1 hybrids were weaker in resource capture and biomass production rate compared with their wild progenitors. However, IMBT F1 hybrids exhibited 90.39% higher aboveground dry biomass than its wild parent. In the current research, the difference in aboveground dry biomass between F1 hybrids, and their wild soybean relatives could be determined by the genotypes of wild soybeans rather than other variation and interactions in environmental or experimental condition. Wild soybeans harbor a high level of genetic variation, and most of the variation was found within the populations and groups, but significant genetic differentiation was also detected among different eco-geographical groups (Dong et al. 2001; Wen et al. 2009; Wang et al. 2014, 2017). Considering the higher level of genetic diversity retained in wild soybeans, this may complicate the consequences of gene flow from transgenic to wild soybeans.

#### Reproductive ability

The reproductive ability of hybrids is one of the most important traits for assessing fitness (Liu et al. 2016). In the present study, reproductive variables of 10 F1 hybrids, including pollen viability and filled seed number per plant, were significantly lower than those of their wild progenitors. Therefore, F1 hybrids may pose less potential ecological risk. Despite all of this, F1 hybrids produced 73–571 filled seeds per plant, and these seeds could, in the next year, germinate seedlings carrying resistant genes, which could survive and produce progenies, especially under glyphosate selection. Moreover, F1 hybrids could backcross with wild progenitors. Thus, the transgenes or other cultivated soybean genes that confer a selective advantage may introgress into wild soybeans.

#### 100-seed weight

The Chinese wild soybean (*Glycine soja*) has three clear genetic categories: small-seeded (100-seed weight under 2.0 g), medium-seeded (100-seed weight of 2.01–2.5 g), and large-seeded (100-seed weight of 2.51–3.0 g). The semi-wild soybean (*Glycine gracilis*) usually had more than 3.0 g 100-seed weight (Wang et al. 2012, 2014). In the current research, six typical small-seed types of wild soybeans with 100-seed weight < 1.5, three small-seed types, JLBC-1, IMBT, and HNSQ (1.6–2.0 g), and one middling seed type JSCZ were used to study the performance of F1 hybrids. The results demonstrated that nine of the 10 F1 hybrids had lower 100-seed weight compared with their wild progenitors. These results differed from those of Guan et al. (2015) and Kan et al. (2015). Two F1 hybrids were approximately 200% more than wild progenitors in the former, and four F1 hybrids were 61–292% more than their wild progenitors in the latter. However, IMBT F1 hybrids had 5.52 g per 100-seed weight, which was 3.56 times that of maternal wild soybean. According to the genetic categories criterion (Wang et al. 2012, 2014), IMBT F1 hybrids should belong to the semi-wild type (*G. gracilis*), which originated from reciprocal hybridization between wild and cultivated soybeans (Wang et al. 2010).

Wang and Li (2011) provided the most direct and convincing evidence that confirmed the natural occurrence of introgression between wild and cultivated soybeans and the hybridization origin of *G. gracilis*. Natural wild-cytoplasmic semi-wild type hybrids (F1) were found between the maternal wild soybeans (collected from Keyouqianqi, Inner Mongolian) and paternal cultivated soybeans. These novel plants produced more seeds with a mean of 4.6 g of 100-seed weight (4.6 times that of wild soybeans). Beside the 100-seed weight, these hybrid plants distinctly differed from their maternal wild soybean populations by having thicker mean diameter for basal stem, higher yield per plant, and aboveground dry mass weight of 18.3 g (5.1 times that of maternal wild soybeans). Similarly, IMBT F1 hybrids had 1.90 times the aboveground dry biomass weight of maternal wild soybeans. The results indicate that gene flow from transgenic soybeans to certain wild soybeans may generate herbicide resistant *G. gracilis*. Therefore, the risk of gene flow from transgenic soybeans to different wild soybean populations must be evaluated before commercial release.

#### Composite fitness

The crossed seed emergence rate, plant height, aboveground dry biomass, fecundity (filled seeds number per plant), and 100-seed weight of F1 hybrids showed greater variability among wild soybeans and transgenic soybeans under benign conditions. The composite fitness ranged from 0.52 to 0.84, with one exception reaching 1.28 across genetic backgrounds. Mercer et al. (2007) found that the hybrid fitness of wild *Helianthus annuus* from different places and cultivated *H. annuus* demonstrated the variable responses to stressful environments. The initial phases of introgression could vary radically in different populations growing under diverse conditions (Mercer et al. 2007). The current results suggest that the introgression from transgenic soybeans into different genotypes of wild soybeans may have different fitness consequences, especially under different condition.

### With weed competition

Weeds may be one of the major constraints to growth of F1 hybrids of wild soybeans and transgenic soybeans in agricultural systems or other system by competing for nutrients, sunlight, space, and water (Renton and Chauhan 2017; Song et al. 2017). Owing to limited crossed seeds, only JLBC-1 F1 hybrids and their progenitors were studied in the presence of weed competition. As expected, the performance of JLBC-1 F1 hybrids and their wild and cultivated progenitors decreased in plant height (F1 hybrid and JLBC-1 had no significant difference), aboveground dry biomass, pod number per plant, filled seeds per plant, and 100-seed weight as well as composite fitness with weed competition (*P* < 0.05). Nonetheless, JLBC-1 F1 hybrids produced 60 filled seeds per plant. From a mechanistic standpoint, the extent of gene introgression depends on the interactions between recombination and selection (Jenczewski et al. 2003). In herbicide-resistant transgenic soybean fields, weeds will be killed and F1 hybrids carrying resistant genes will survive under herbicide selection. Therefore, once the initial gene flow occurred through pollen from transgenic to wild soybeans, the F1 hybrids could survive and complete their life cycle in the face of either weed competition or herbicide selection.

## Conclusion

Without weed competition, compared with their respective wild soybean relatives, crossed seeds obtained from wild soybeans as maternal plants and glyphosate-resistant transgenic soybean as paternal plants emerged at low rates and the composite fitness of nine F1 hybrids was significantly lower except for IMBT F1 hybrid that had similar fitness to its wild progenitor. With weed competition, JLBC-1 F1 hybrids displayed lower fitness than their wild soybean relative. However, all F1 hybrids produced approximately 70–500 filled seeds/plant in absence of weed competition. Moreover, two thirds of JLBC-1 F1 hybrids survived, and produced 60 filled seeds/plant under weed competition. The results implied that F1 hybrids may establish in nature environment.

## Electronic supplementary material

Below is the link to the electronic supplementary material.

Supplementary file1 (DOCX 3329 kb)
