# Supplementary material for: Fitness of F1 hybrids between 10 maternal wild soybean populations and transgenic soybean
Source: Transgenic Res. 2021 Jan 5;30(1):105–19. doi: 10.1007/s11248-020-00230-x (PMC7854435; doi:10.1007/s11248-020-00230-x)
Supplement: Supplementary file 1 — Supplementary file1 (DOCX 3329 kb) [file 11248_2020_230_MOESM1_ESM.docx]

**Supplementary Table 1 Seeds number of wild soybeans and F1 hybrids sowed in the experiments**

| Wild soybean | Number | F1 Hybrid | Number |
| --- | --- | --- | --- |
|  | 2017 2018 2019 |  | 2017 2018 2019 |
| HLJHRB-1 | 100 － － | HLJHRB-1 F1 | 100 － － |
| JLBC-1 | 100 － － | JLBC-1 F1 | 120 － － |
| LNTL | 100 － － | LNTL F1 | 50 － － |
| JSCZ | 100 － － | JSCZ F1 | 30 － － |
| HLJHRB-2 | － 100 － | HLJHRB-2 F1 | － 75 － |
| JLBC-2 | － 100 － | JLBC-2 F1 | － 75 － |
| LNSY | － 100 － | LNSY F1 | － 75 － |
| HBHD | － 100 － | HBHD F1 | － 75 － |
| IMBT | － － 100 | IMBT F1 | － － 30 |
| HNSQ | － － 100 | HNSQ F1 | － － 30 |

**Supplementary Table 2 Performance of transgenic soybean in three years**

**(mean ± SE)**

| Variables | Year | | |
| --- | --- | --- | --- |
|  | 2017 | 2018 | 2019 |
| Emergence rate（%） | 93.00±3.39 | 85.00±3.54 | 86.00±3.32 |
| Length of cotyledons（mm） | 20.11±0.25 | 19.73±0.35 | 20.67±0.22 |
| width of cotyledons（mm） | 11.41±0.17 | 11.51±0.18 | 11.72±0.15 |
| Length of true leaves（mm） | 32.09±0.50 | 30.71±0.75 | 37.93±0.67 |
| width of true leaves (mm) | 28.42±0.48 | 25.22±0.80 | 33.81±0.41 |
| Plant height（cm） | 31.11±0.67 | 32.43±0.84 | 19.83±0.39 |
| Aboveground dry biomass（g） | 129.53±7.77 | 142.37±4.36 | 85.18±4.88 |
| Pod number/plant | 83.80±2.06 | 99.45±4.12 | 74.13±6.56 |
| Filled seeds/plant | 210.37±8.93 | 239.15±11.95 | 165.87±14.37 |
| 100-seed weight（g） | 18.28±0.06 | 17.85±0.39 | 18.27±0.16 |


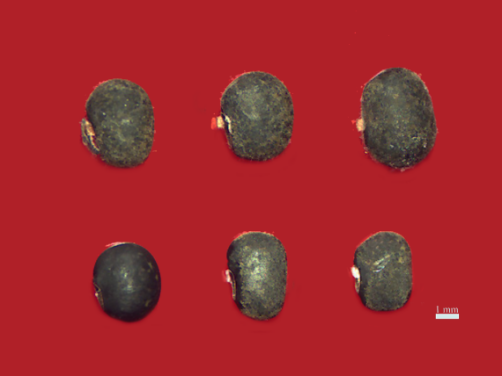

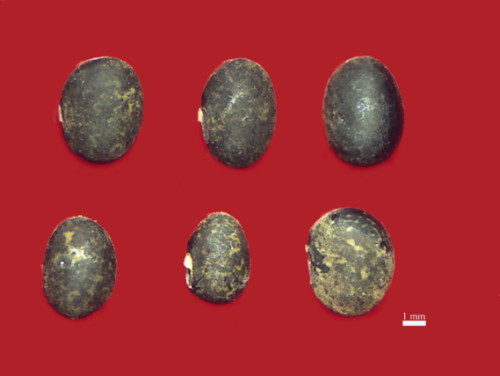


JLBC-1

HLJHRB-1


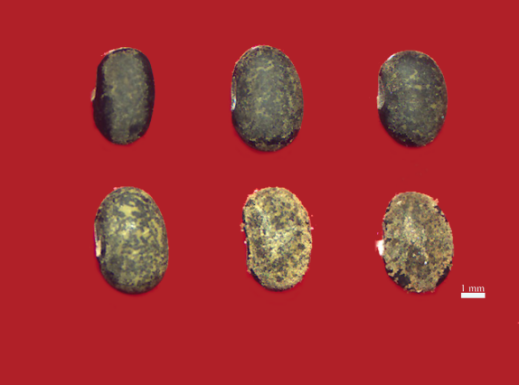

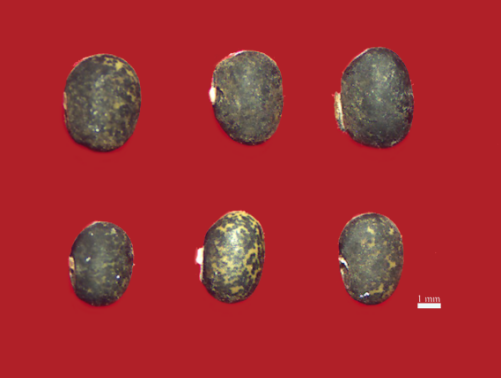


LNTL

JSCZ


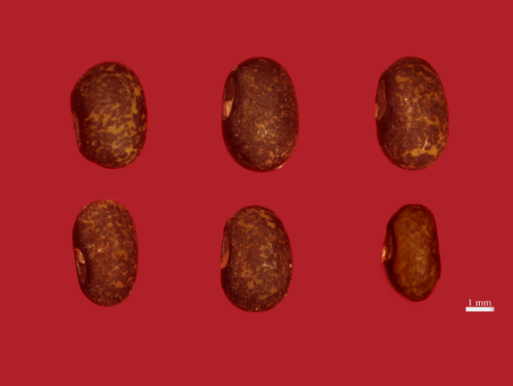

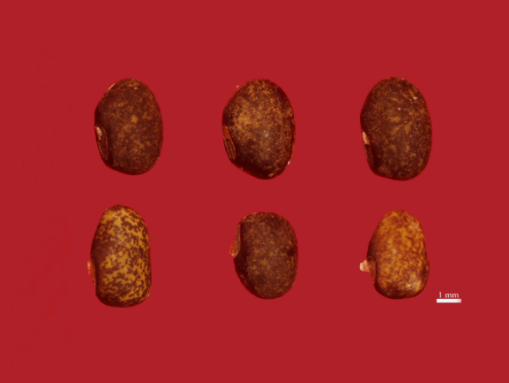


JLBC-2

HLJHRB-2

**
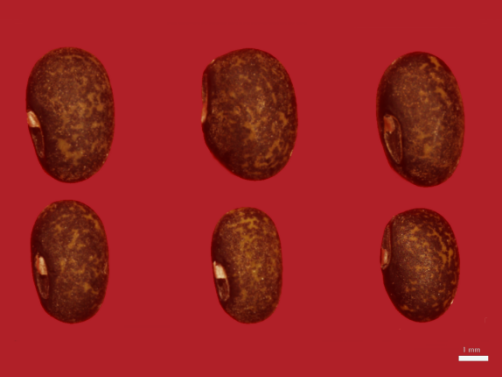

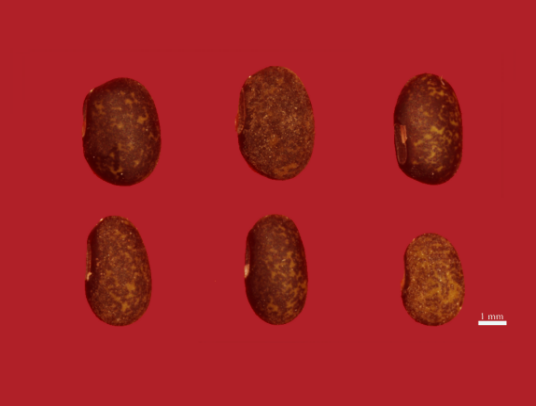
**

HBHD

LNSY

**
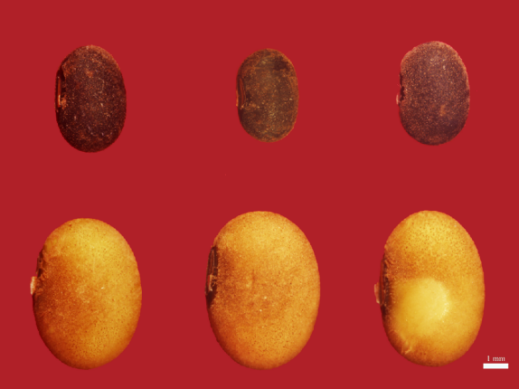

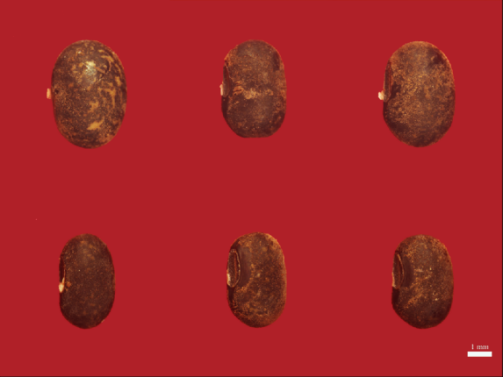
**

IMBT

HNSQ

**Note：**The first row was selfed seeds of wild soybean.

The second row was selfed seeds of F1 hybrid.


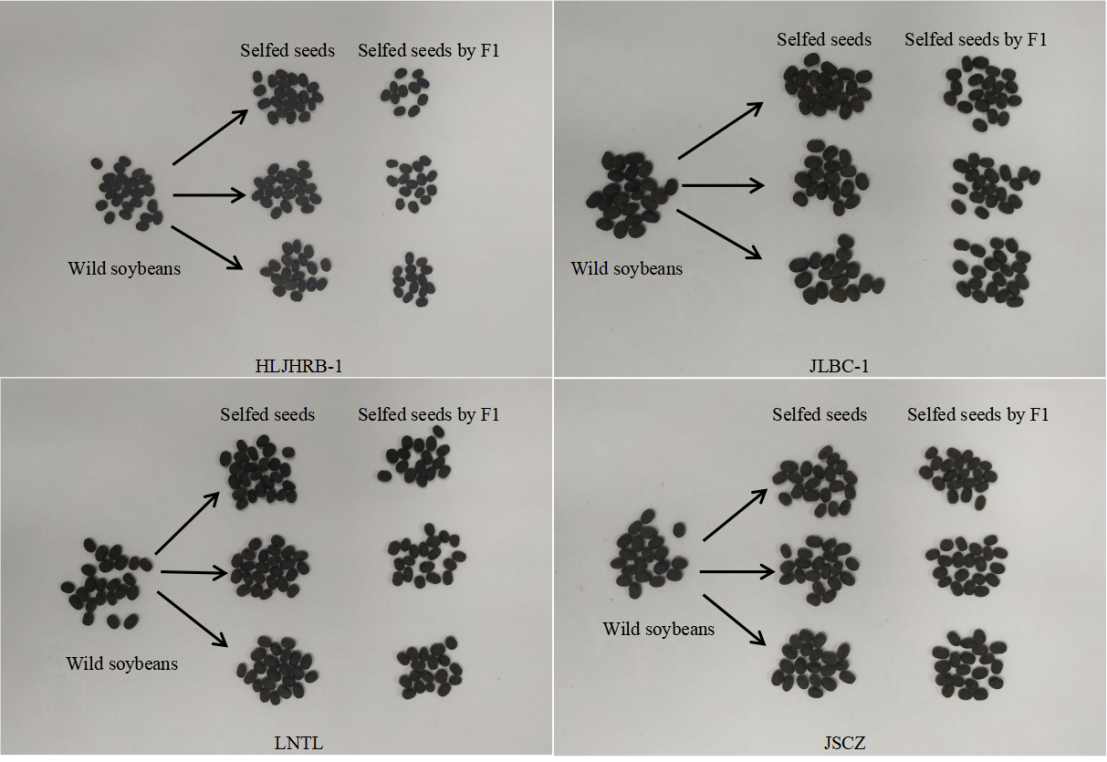


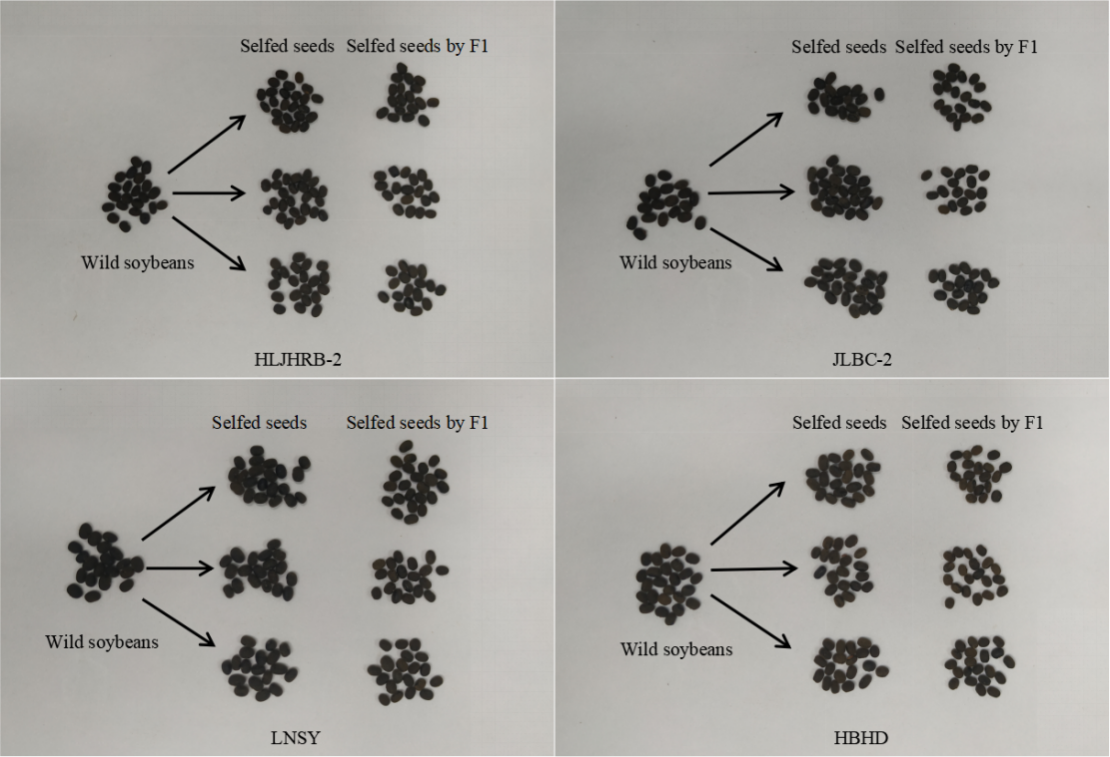


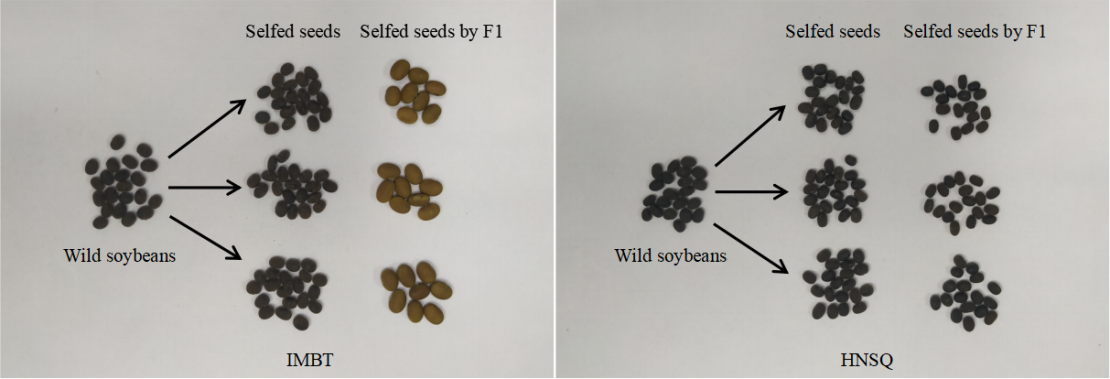


**S**upplementary Figure 1. Selfed seeds of wild soybean and its F1 hybrid
